# Protocol for evaluating external facilitation as a strategy to nationally implement a novel stigma reduction training tool for healthcare providers

**DOI:** 10.1186/s43058-022-00332-z

**Published:** 2022-08-12

**Authors:** Sally Wasmuth, Johnna Belkiewitz, Dawn Bravata, Caitlin Horsford, Alex Harris, Carlton Smith, Charles Austin, Edward Miech

**Affiliations:** 1 Department of Occupational Therapy, School of Health and Human Sciences, Indiana University Purdue University Indianapolis, 306 Coleman Hall, 1140 W. Michigan Street, Indianapolis, IN 46202 USA; 2 HSR&D Center for Health Information and Communication (CHIC), Health Services Research and Development (11H), Richard L. Roudebush VA Medical Center, 1481 W. 10th St, Indianapolis, IN 46202 USA

**Keywords:** Implementation climate, External facilitation, Stigma reduction, Diversity training, Healthcare equity, Theatre-based interventions, Influencing factors, Occupational therapy, Occupational justice, Consolidated Framework for Implementation Research

## Abstract

**Background:**

Identity Development Evolution and Sharing (IDEAS) is a theatre-based intervention for reducing healthcare provider stigma. IDEAS films are created by collecting narratives from people who have experienced discrimination and healthcare inequity, partnering with professional playwrights to create theatrical scripts that maintain the words of the narratives while arranging them into compelling storylines involving several interviews, and hiring professional actors to perform and record scenes. IDEAS implementation requires a moderator to establish a respectful learning environment, play the filmed performance, set ground rules for discussion, and moderate a discussion between healthcare providers who viewed the film and invited panelists who are members of the minoritized population being discussed. IDEAS’ impact on provider stigma is measured via pre/post Acceptance and Action Questionnaire – Stigma (AAQ-S) data collected from participating providers. The objectives of this manuscript are to provide narrative review of how provider stigma may lead to healthcare inequity and health disparities, describe the conceptual frameworks underpinning the IDEAS intervention, and outline methods for IDEAS implementation and implementation evaluation.

**Methods:**

This manuscript describes a hybrid type 3 design study protocol that uses the Consolidated Framework for Implementation Research (CFIR) to evaluate external facilitation, used as an implementation strategy to expand the reach of IDEAS. CFIR is also used to assess the impact of characteristics of the intervention and implementation climate on implementation success. Implementation success is defined by intervention feasibility and acceptability as well as self-efficacy of internal facilitators. This manuscript details the protocol for collection and evaluation of implementation data alongside that of effectiveness data. The manuscript provides new information about the use of configurational analysis, which uses Boolean algebra to analyze pathways to implementation success considering each variable, within and across diverse clinical sites across the USA.

**Discussion:**

The significance of this protocol is that it outlines important information for future hybrid type 3 designs wishing to incorporate configurational analyses and/or studies using behavioral or atypical, complex, innovative interventions. The current lack of evidence supporting occupational justice-focused interventions and the strong evidence of stigma influencing health inequities underscore the necessity for the IDEAS intervention.

Contributions to the literature

- This paper details a novel theatre-based training tool to reduce implicit provider bias to promote healthcare equity; this is critical given the vast healthcare inequities impacting the wellbeing of minoritized populations.
- The methods for evaluating implementation success described in this study add unique contributions to the literature by describing how configurational analyses can be used to explore how complex factors, such as culture and site priorities, influence implementation, and impact of interventions.
- By outlining a process for identification of moderating variables, healthcare teams can be better equipped to strategically implement innovative initiatives, such as stigma reduction and diversity-focused trainings.

## Background

The field of occupational therapy is committed to fostering a culture of occupational justice and supporting the wellbeing of clients [1]. Occupational justice is “the right of every individual to be able to meet basic needs and to have equal opportunities and life chances to reach toward their potential…specific to the individual’s engagement in diverse and meaningful occupations” [2]. The field also emphasizes the crucial role of service, system, and policy structure at a community and society level to either promote or inhibit this access to justice [1]. A critical barrier to progress in occupational therapists’ ability to promote equity in health care is the limited availability of evidence-based approaches occupational therapists can implement to support occupational justice. To address this gap, external facilitation will be used to disseminate Identity Development Evolution and Sharing (IDEAS), a program for reducing healthcare provider stigma, with a growing body of supporting evidence [3, 4]. This manuscript describes (1) the IDEAS intervention, (2) the planned implementation and implementation evaluation; and (3) provides a narrative review of how provider stigma may lead to healthcare inequity and health disparities.

### Intervention: Identity Development Evolution and Sharing (IDEAS)

IDEAS is a one-hour, client-centered, occupation-based, theatre intervention to reduce stigma beliefs among healthcare providers [3, 4]. IDEAS performances are created by conducting narrative interviews with people from a target population who have been harmed by provider stigma and translating those narratives into a theatrical performance to be performed by professional actors for healthcare providers. IDEAS implementation requires a moderator to establish a respectful learning environment, play the filmed performance, set ground rules for discussion, and moderate a discussion between healthcare providers who viewed the film and invited panelists who are members of the minoritized population being discussed. The three existing IDEAS videos to be used in this study are as follow: “The Rest of my Life”—an account of several peoples’ journeys with substance use disorder (SUD)—“Stories of Inequity”—ten Black women’s accounts of experiencing discrimination in healthcare—and “This Authentic Person”—the stories of several transgender and gender-diverse people’s journeys with gender and societal stigma.

IDEAS is rooted in a robust body of literature that suggests attending to stories through literature, art, theatre, and poetry improves clinical encounters and interpersonal relationships; this attention increases empathy and understanding [5–8]. Clinicians become better able to notice important nuances of people’s stories. Inter-professional communication improves [5, 8]. Fewer medical errors are made. Psychological flexibility increases. An IDEAS performance is an opportunity to attune to and witness others’ stories. Attending to stories through theatre provides viewers with an opportunity to privately witness and confront their own implicit biases [3, 4]. By becoming aware of and grappling with one’s own biases, viewers gain flexibility regarding how they respond to those biases in the moment with clients, co-workers, and people in general [9]. By contrast, being unaware of one’s own personal biases can result in problematic interpersonal interactions such as committing microaggressions, embodying a stigmatizing or alienating demeanor, and/or making inequitable, harmful decisions regarding client care [10].

### Conceptual models

#### Conceptual framework for evaluating IDEAS effectiveness on stigma reduction and enhanced psychological flexibility

The primary quantitative outcome measure will be the AAQ-S, which measures *enacted*, stigma, i.e., stigma beliefs that produce actions such as discriminating against people or groups [9]. Levin and colleagues [9], drawing on the work of Akrami, Ekehammar, and Bergh [11], conceptualize stigma as “a more general tendency to evaluate and discriminate against others based on their group membership, rather than being specific to attitudes towards any one group in particular.”^9(p21)^ The AAQ-S was created through the lens of Acceptance and Commitment Therapy (ACT) and is rooted in the notion that all people exist within shared contexts of racism and other forms of discrimination at the individual, group, and institutional level and are thus affected by stigma whether beliefs of personal biases exist or not [9, 12, 13]. Implicit biases often contradict personal values, which lends insight as to how medical providers who value equitable health care provision can make inequitable decisions that cause harm to those seeking care. Psychological flexibility is “the capacity to actively embrace one’s private experiences in the present moment and engage or disengage in patterns of behavior in the service of chosen values.” [9] ^(p21)^ The greater the psychological flexibility a person has regarding their own biases, the more choices they have in terms of how to respond to discriminatory thoughts or ideas [9]. The ability to recognize and distance one’s self from their own biases is critical to providing equitable health care. The AAQ-S is a reliable and valid measure of stigma and psychological flexibility [9]. The AAQ-S has good inter-rater reliability (Cronbach’s alpha = 0.84) and low to moderate correlations (*r* = 0.23—0.43) with measures of right-wing authoritarianism, social dominance, ethnocultural empathy, empathic concern, and perspective taking [5, 9].

#### Central characteristics of psychological flexibility measured by AAQ-S [9] ^(p22)^

**Table:** 

Flexible awareness of one’s private experiences in the present moment, including stigmatizing thoughts	
De-fusion from stigmatizing thoughts (seeing thoughts as just thoughts rather than something literally true)	
Willingness to have stigmatizing thoughts, rather than engaging in ineffective forms of avoidance (e.g., thought suppression, avoiding situations where stigmatizing thoughts occur)	
Relating to oneself and others as distinct from thoughts and feelings about them	
Clarifying valued patterns of activity in social interactions	
Committing to patterns of valued activity with others, even when stigmatizing thoughts and feelings seem to stand in the way	

#### Conceptual framework for evaluating implementation

The Consolidated Framework for Implementation Research (CFIR) [14] will be used; this tool is a widely-used framework with over 2,000 citations in PubMed and over 5500 Google Scholar citations designed to “identify factors that might influence intervention implementation and effectiveness.” [15] ^(p15)^ Several CFIR resources will be used, including the CFIR interview generator, and guidelines for qualitative coding and quantitative rating of each of the CFIR implementation constructs on which data will be collected (see Table 1).

**Table 1:** CFIR constructs

Intervention characteristics	
**Relative advantage:** How does the intervention compare to other similar existing programs in your setting or to other alternatives that may have been considered? Is there another intervention that people would rather implement? **Design and quality packaging:** What is your perception of the quality of the supporting materials, packaging, and bundling of the intervention for implementation? 1. What supports, such as online resources, marketing materials or a toolkit, are available to help you implement and use the intervention? How will available materials affect implementation in your setting?	
Inner setting	
**Culture:** How would you describe the culture of your organization? Of your own setting or unit? How do you think your organization’s culture (general beliefs, values, assumptions that people embrace) will affect the implementation of the intervention? To what extent are new ideas embraced and used to make improvements in your organization **Implementation climate:** What is the general level of receptivity in your organization to implementing the intervention? **Tension for change:** Is there a strong need for this intervention? How essential is this intervention to meet the needs of the individuals served by your organization or other organizational goals and objectives? How do people feel about current programs/practices/process that are available related to the intervention? **Compatibility:** How well does the intervention fit with your values and norms and the values and norms within the organization? How well does the intervention fit with existing work processes and practices in your setting? Can you describe how the intervention will be integrated into current processes? Will the intervention replace or compliment a current program or process? **Relative priority:** Describe activities or initiatives that (appear to) have highest priority for you (for the organization).	
Implementation success outcomes	
**Executing:** Has the intervention been implemented according to the implementation plan? Why or why not? **External facilitation:** Was the external facilitator approachable? In what ways and to what extent was the external facilitator available to you (e.g., set office hours, on call, available by phone, email, text, other?) Were these modes of communication sufficient? Would in-person facilitation have been preferred? Why or why not? How often did you solicit external facilitation support? How often was it offered without solicitation? Should it have been offered more or less frequently? Why or why not? **Self-efficacy:** How confident are you that you will be able to successfully implement (post-implementation: were you able to successfully implement) the intervention? Why? How confident do you think your colleagues feel about using the knowledge they will gain (post-implementation: gained) from the intervention?	

## Methods

### Aims

The overall approach will involve (1) evaluating external facilitation to expand IDEAS implementation across multiple occupational therapy clinical sites and (2) measuring the effect of IDEAS on stigma beliefs of practicing occupational therapists.

### Participants

Convenience and chain sampling will be used to recruit up to ten implementing occupational therapists—internal facilitators—to lead the IDEAS intervention within their respective clinics. Participants will intentionally be recruited from a diverse range of sites with respect to geographic location and clinical setting type (e.g., pediatric, skilled nursing, inpatient rehabilitation). In addition to the ten implementing occupational therapists who will be internal facilitators, participants of this study will include the staff occupational therapists within the clinic who participate in the IDEAS training and clinical managers who participate in interviews to provide information about the implementation climate.

### Settings

Partnering sites will include a variety of urban, suburban, and rural occupational therapy clinical settings that serve a wide range of patients across the lifespan. Confirmed sites include outpatient pediatric clinics, adult inpatient rehabilitation hospitals, and extended care facilities. Sites will vary from smaller occupational therapist-led clinics to occupational therapy departments within large hospital networks. IDEAS is implemented as a group training for staff occupational therapy practitioners via a combination of in-person and virtual interactions. The initial training of the internal facilitator occurs via zoom. The IDEAS implementation can occur during an in-person or Microsoft Teams staff meeting. If staff meeting is in-person, expert panel speakers join the meeting via zoom or teams on a screen in the meeting room. If virtual, the panel speakers join the staff on the virtual platform.

### Study design

Using a hybrid type 3 design, a formative evaluation of IDEAS implementation will be conducted using external facilitation as the implementation strategy [16, 17]. Evaluative objectives are to (1) collect and analyze quantitative data on provider (occupational therapists IDEAS participants) pre/post stigma beliefs using the AAQ-S; and (2) collect qualitative pre/post data from IDEAS participants, implementors, and clinical managers regarding their experiences of implementation within their site. The latter will be analyzed using CFIR analysis guidelines (described below).

### Implementation strategy—external facilitation

The external facilitator (SW) will support occupational therapists/internal facilitators from diverse practice settings throughout the USA in implementing a single IDEAS intervention within their local clinical sites via a one-time external facilitation virtual meeting. During this meeting, the external facilitator will provide and review with each implementing occupational therapist/internal facilitator an e-resource manual (see Appendices 1 and 2), three IDEAS performance video links (sites select which performance is the best fit with respect to their priorities), and a list of contact information for panelist speakers for each video. Following the initial external facilitation meetings, the external facilitator will provide support via phone, text, and email exchanges as needed. Dose of external facilitation will be tracked via an external facilitation tracking log in which the external facilitator notes type of communication (email, phone, zoom), frequency, date, and duration of each encounter.

### Implementation success

Our primary measures of implementation success are self-efficacy of implementors, experiences of external facilitation, and feasibility and acceptability of IDEAS [11, 18, 19].

### Data collection

The research team (excluding the external facilitator) will conduct 3 pre/post stakeholder interviews at each implementation site; one with the implementing occupational therapist/internal facilitator, one with a clinical manager, and one with an occupational therapy practitioner who intends to participate in the IDEAS training. These interviews are created using the CFIR interview generator and include the constructs listed in Table 1 [20].

Following the initial external facilitation meeting between the external and internal facilitators, the internal facilitator will complete an online survey containing the items of the Acceptability of Intervention Measure (AIM), the Intervention Appropriateness Measure (IAM), and the Feasibility of Intervention Measure (FIM) regarding their experience of external facilitation [21]. Following IDEAS implementation, the internal facilitator and the occupational therapist who participate in both the IDEAS training and stakeholder interview will complete the AIM and IAM questions regarding the intervention itself. The survey questions are listed in Table 2 and scored on a 5-point scale from (1) “completely disagree” to (4) “completely agree.”

**Table 2:** IDEAS post-implementation survey

Intervention (IDEAS) acceptability	
**Acceptability of Intervention Measure (AIM)** • IDEAS meets my approval. • IDEAS is appealing to me. • I like IDEAS. • I welcome IDEAS. **Intervention Appropriateness Measure (IAM)** • IDEAS seems fitting. • IDEAS seems suitable. • IDEAS seems applicable. • IDEAS seems like a good match. **Feasibility of Intervention Measure (FIM)** • IDEAS seems implementable. • IDEAS seems possible. • IDEAS seems doable. • IDEAS seems easy to use.	
Implementation approach (external facilitation) acceptability measures completed by therapist stakeholders	
**Acceptability of Intervention Measure (AIM)** • The External Facilitator’s facilitation approach meets my approval. • The External Facilitator’s facilitation approach is appealing to me. • I like the External Facilitator’s facilitation approach. • I welcome the External Facilitator’s facilitation approach. **Intervention Appropriateness Measure (IAM)** • The External Facilitator’s facilitation approach seems fitting. • The External Facilitator’s facilitation approach seems suitable. • The External Facilitator’s facilitation approach seems applicable. • The External Facilitator’s facilitation approach seems like a good match.	

Occupational therapists who attend the IDEAS training will complete pre/post AAQ-S surveys electronically; the pre-survey also contains demographic questions such as age, ethnicity, race, and whether the person identifies as a member of the minoritized population the IDEAS intervention is focused on (yes, no), as well as a brief description of the study and a space for providing written informed consent.

### Analytical methods

#### Evaluating implementation

Members of the research team will use the CFIR codebook template, which contains CFIR definitions and coding guidelines, to code each stakeholder interview from 10 implementation sites. The team will highlight data corresponding with each of the constructs listed in Table 1, while remaining open to additional emerging codes as needed to highlight pertinent data related to implementation. The team will work together on coding until consistent agreement is reached on how to code the data. The team then will code remaining transcripts in pairs, returning to prior transcripts and recoding as needed if/when the codebook is revised.

Following qualitative coding, the team will independently review the data for a single site and meet to assign a quantitative rating to each code based on qualitative data from that site, rating from − 2 to +2 based on whether each construct has a positive or negative impact on implementation success. For example, if the packaging of the intervention is described as problematically interfering with successful implementation (e.g., the film does not work), that construct of “quality packaging” will receive a − 2 rating. If the packaging is problematic (the film quality is poor but still works), the construct of “quality packaging” will receive a − 1. If the packaging does not influence implementation, it will receive a 0, and if it has a positive or highly positive impact on implementation, it will receive a + 1 or + 2, respectively. The team will enter their ratings in a blinded vote and will discuss any discrepancies, re-voting until consensus is reached for each construct.

Implementation success will be rated as high or low, based on average scores from the AIM, IAM, and FIM items as well as consensus ratings on the CFIR constructs of self-efficacy and experiences of external facilitation [14, 21]. Configurational analysis will then be conducted to determine difference-making combinations of CFIR-related conditions uniquely distinguishing higher- from lower-performing sites. Configurational analysis will draw upon Boolean algebra and set theory to identify a “minimal theory” or a crucial set of difference-making combinations that remove redundancies and that uniquely distinguish one group of cases from another (i.e., those with versus those without implementation success) [22–28]. Major strengths of this approach include its ability to model equifinality, when multiple paths lead to the same outcome; its capacity to identify complex relationships, when several conditions work together jointly as a whole; and its versatility with small studies [29–33]. Configurational analysis in this project will be conducted using Coincidence Analysis (cna) and is supported by using the R package “cna” as well as the software applications R and R Studio.

#### IDEAS effectiveness on provider stigma and correlations with implementation success

IDEAS effectiveness will be measured regarding its impact on provider stigma using repeated measures ANOVA to compare average provider AAQ-S pre-post change scores within and between sites [9]. A priori power analysis will be performed to determine the required sample size needed to obtain adequate statistical power for the IDEAS intervention. Using the following parameters based on repeated measure ANOVA within- and between- factors of two measurement time points, it assumes effect size of 0.80, *α* at 0.05, *β* at 0.80, and the correlation between repeated measures of 0.5. The required total sample size is determined to be 80. The aim, therefore, is to have 8 occupational therapist complete IDEAS, including the pre/post AAQ-S, at 10 sites. Effectiveness outcomes will be included in configurational analyses described above to explore whether/how implementation success and associated factors are related to effectiveness. For example, it is hypothesized that observations of high implementor self-efficacy combined with organizational cultures that embrace change may correlate with greater intervention effectiveness at that site, demonstrated via larger AAQ-S changes scores [9].

## Discussion

### Literature review of provider stigma, healthcare inequity, and health disparities

This project is supported by an overwhelming body of literature demonstrating (1) the degree to which provider biases detrimentally affect health outcomes for marginalized groups; (2) the limited evidence-based occupational justice interventions despite the profession’s commitment to client-centered practice, advocacy, and equity; and (3) the lack of adequate measures for examining provider bias on clients’ healthcare experiences [1, 6, 34, 35]. Existing reliable assessments for examining provider bias mainly rely on self-report measures with only a few contextualizing the practitioner within the workplace to include structural and systematic factors [36–40].

### Health inequities and disparities resulting from stigma and bias

Numerous studies have illustrated how a longstanding history of systemic racism has influenced health disparities [41, 42]. The American Heart Association has linked poor cardiovascular health to self-reported experiences of racism, and literature has documented poor health outcomes for Black women compared to White women in many areas, including but not limited to pre/post-natal, mental health, and pain outcomes [43, 44]. This has been especially true for Black women with a history of substance misuse [45, 46]. Health disparities for gender minorities are alarming as well. The 2015 U.S. Transgender Survey report revealed that transgender and gender diverse (TGD) individuals (1) avoid needed medical services because they have been vastly mistreated in healthcare settings and (2) experience grave occupational injustices in society such as lacking access to restrooms and experiencing exorbitant rates of physical and sexual violence because of their gender identity and/or presentation [6]. Fear of discrimination has been linked to higher rates of anxiety, depression, suicide attempts, social isolation, and occupational marginalization [47]. Moreover, fear of discrimination during healthcare encounters reduces health behaviors to prevent and manage chronic conditions [6]. In addition, stigmatized health conditions such as substance use disorder (SUD) continue to plague the United States despite numerous accessible, evidence-based practices. Literature highlights the impact of stigma on substance users’ utilization of healthcare and recovery services, treatment engagement, and willingness to be honest with providers [34].

### Novel use of an existing evidence-based approach: advantages over other methods

The importance and innovation of IDEAS as an occupational therapy intervention lie in its ability to evoke empathy, understanding, and awareness in audience members by connecting them not to abstract, dehumanized stories, but rather to the stories of people present during IDEAS as panel speakers. Literature suggests that explicit bias is largely absent in health care, but that implicit biases continue to drastically impede health outcomes for marginalized groups [42]. Meineke [4], through a cognitive science lens, describes how and why theatre-based approaches may succeed in reducing implicit biases of audiences. The team’s prior work lends further evidence to Meineke’s findings; in prior studies of IDEAS, a significant decrease in stigma beliefs of healthcare providers was found from baseline (*M* = 79.93, *SD* = 12.62) to follow-up (*M* = 69.74, *SD* = 12.77) measured by the Acceptance and Action Questionnaire—Stigma (AAQ-S); *t*(41) = 8.18, *p* < 0.0001, *d* = 0.80 [9]. The IDEAS intervention is advantageous compared to other stigma reduction approaches within health care because it can engage and affect its viewers more readily than standard cultural competence training programs. Its use of narrative theatre, which offers audiences an opportunity to immerse themselves in the stories of others and develop cultural humility, can cultivate open-mindedness and changed attitudes where more overtly persuasive messaging triggers cognitive resistance [8, 48, 49].

### Lack of adequate measures of provider bias

This implementation research provides a critical outline for evaluating the effects of moderating variables on provider bias-targeted trainings, as measures detailed in existing literature examine provider bias within a limited scope by relying heavily on uncontextualized self-report assessments. Self-report and some client-report measures have been utilized within healthcare provider cultural competence education; however, these assessments lack recognition of the important role that environmental influences play into practitioner bias and stigma-influenced care provision [35]. Though “organizational support” has been evidenced as a distinguished and critical factor in the measurement of cultural competence, it is not widely validated in existing healthcare provider bias and stigma-related assessments [36–40].

### Limitations

The COVID-19 pandemic has affected site recruitment and implementation of the IDEAS intervention. Several larger hospital networks were established as initial partners; however, with the frequent spikes in COVID-19 patient numbers, these sites retracted from their original timeline for the program, citing the pandemic as higher priority for administration and management. As the goal is to include a diverse cohort of healthcare facilities from across the country, this has restricted or delayed some of the site recruitment options for the IDEAS intervention.

### Future study

The resources shared with the internal facilitator provide a checklist for how to deliver IDEAS. We plan to track lessons learned from IDEAS implementation in this study as well as any adaptations or unanticipated changes/alterations that occur and use this information to determine which elements of IDEAS implementation are critical for implementation success. Data will consist of personal communications and observations as well as CFIR post-interview data [9]. These data will inform eventual development of a fidelity scale to allow for future fidelity assessment.

## Data Availability

The datasets used and/or analyzed during the current study are available from the corresponding author on reasonable request.

## Appendix 1

Table 3

**Table 3:** IDEAS implementation E-resource manual for internal facilitator

Important contact information	
**Principal investigator** Available by text, phone call, or email: **Project manager** 2. Available by email:	
3. Intervention fidelity checklist for implementing OT	
**Prior to:** **▪ Decide on IDEAS implementation date and provide to project manager** **▪ Provide stakeholder names and contact information (1 manager, 1 OT trainee)** **▪ Provide mailing address and confirm receipt of shared assumptions and agreements (received via mail**—**hardcopy, electronic back-up)** **▪ Provide e-flyer: pre/post-test links, site code, and shared assumptions and agreements to OTs** **▪ Provide call-in details for research team member and panelists; ensure appropriate equipment** **▪ Confirm scheduled panelists with project manager** **During:** **▪ Take attendance and inquire about/document reasons for absences** **▪ Remind people of pre-test/post-test** **▪ Provide transparent introduction regarding role as the facilitator and capacity for that role** Discuss limitations, special skills, and boundaries of the role. Below is an example of what I might personally say when discussing my skills, limitations, and boundaries. You will need to think through what to say regarding your own skills and limitations. You can read the ‘Role’ verbatim or adjust the wording as you see fit while keeping with the general tenets described below. Skills: I have a background in African American studies and have been doing participatory research in the community focusing on addiction and mental health recovery for the past 10 years. I have been researching arts-based initiatives to enhance meaningful participation and reduce implicit provider bias, stigma, and the harmful impacts of anti-black racism for the past 5 years. I have training as an OT and in facilitating supportive educational and/or therapeutic groups. Limitations: While I have had many opportunities to participate in and lead this work, my experience is also inseparable from my experiences of privilege in the world as a cis-gender, white woman. I recognize it is critical for me to continuously seek opportunities for increased growth and understanding on ways to help combat the harms that have resulted from centuries of oppression directly related to this country’s history. Role: My role today is both supported and limited by my own personal experiences—I am here as a facilitator, to hold a space for us to engage in this reflective work of self-discovery, and to encourage continued work as well as the everyday use of what is learned today. My role is to walk us through today’s training, and to ensure that we recognize and uphold a set of shared agreements to promote a safe and supportive space. **▪ Verbally review shared assumptions and shared agreements; ask group to raise hand in agreement to uphold these throughout** **▪ Explain what IDEAS is and isn’t** o IDEAS is an opportunity to attune to/witness others’ stories in order to improve clinical encounters through increased empathy and understanding, and enhanced awareness of personal biases. Watching IDEAS performances allows us to observe our own reactions and grapple with them on our own time so that we have more flexibility with how we respond in the moment with clients. o IDEAS is NOT a training tool that teaches particular skills or approaches for working with client populations, although performances may contain preferred terminology for addressing certain populations and informational/educational content about the impacts of stigma and institutional racism. o IDEAS is an adapted, theatre-based intervention (we are not film-makers!) IDEAS performances consist of actors performing the verbatim words pulled from narrative interviews. **▪ Acknowledge differences in the room and provide trigger warning** o Trigger warning: It is important to note there is some content that may be distressing or triggering for some people in the room—this performance may contain content related to drug use, systemic violence, microaggressions, trauma, and loss. **▪ Play full film** **▪ Zoom in 2**–**3 panelists and moderate panel discussion** o First, have the panelists introduce themselves o Optional starter questions: **▪** What stood out the most in the film? **▪** What surprised you? **▪** What questions do you have about the film’s content? **▪ Uphold assumptions/agreements** **After** **▪ Email number of attendees and any known reasons for absences to program director** **▪ Ensure receipt of e-gift-cards and distribute to all attendees** **▪ Encourage post-test completion**	
IDEAS performances THIS AUTHENTIC PERSON: Stories from transgender and gender diverse people STORIES OF INEQUITY: Black women’s experiences of discrimination in healthcare I’M DOING THIS FOR THE REST OF MY LIFE: Stories from people with substance use disorders and other addictive behaviors	

## Appendix 2 Shared assumptions

*Adapted from* *dismantlingracism.org*

- No one is exempt from the toxicity of institutional racism, ableism, classism, cis-genderism, heterosexism, colonialism, and ethnocentrism, and that have pervaded our culture
- People have been discouraged from seeing the toxicity of our culture; we therefore must take actions to *learn* to see our culture and how grave inequities have come to be perceived as normal (but should not be!)
- We can have good intentions and still have a hurtful or damaging impact
- Unlearning oppressive attitudes will take a lifetime. Most of us have been struggling with these issues for years and years already. None of us are beginners and none of us have perfect clarity. This work is a journey without endpoint. This work is a lifelong process.
- We operate in a culture that assumes justice, equity, and civil rights efforts are "for" non-majority communities and people and that the goal is to “help them”, which perpetuates the notion that the majority population is the default “normal” and doesn’t need to change. We believe this worldview perpetuates the problem(s).
- We are not here to “fix” each other; rather our work is to love ourselves into who we are, knowing how conditioned we all are by white supremacy.
- We have to believe in the possibilities of creating the world we want to see by walking our talk and learning from our mistakes.
- None of this is easy and we have to do it anyway.

Shared agreements

*Adapted from* *nonprofitaf.com*

Let us provide feedback on actions and opinions, not motivation or character: In this sort of environment, it is easy to lose focus on ideas and actions; instead, we start questioning people’s motivations and attacking their character. Instead of “I disagree with you on blah blah,” it becomes “You only say blah blah because you are blah blah and you want blah blah because all you blah blahs are so-and-so!” Let us check ourselves when we do that. It never does any good. People never respond well when their character or motivations are being questioned. It shuts down conversations, or worse, generates in-kind retaliation and perpetuates a vicious cycle.

Let us assume the best intentions, but also address impact: In trying to be helpful, many of us say and do things that may not end up very helpful. Let us assume everyone means well and let us be patient with one another. But that does not override the fact that our well-intentioned actions may be hurtful to others. When our actions are damaging, we must address them, whether or not they were well-intended.

Let us not assume we completely understand one another’s reality: Right now, many communities are hurting. Latino kids are getting “Build that wall!” chanted at them. Women wearing hijabs are attacked, or threatened to be set on fire. Black university students are getting pictures of lynchings texted to them. Many of people from marginalized communities do not feel safe anymore, and some of us are not sure we will ever be able to. If we do not come from these communities, let us not assume there is an equivalence with our own background. Let us not assume we know what another person is going through. I have seen too many conversations where people are dismissive of one another’s experiences. Let us agree not to do that.

Let us be gracious in accepting difficult feedback: When someone gives us feedback, especially those who come from backgrounds completely different from ours, it is a valuable gift. And it often comes with a price for the person giving the feedback, as they frequently must share vulnerable personal information in order to bring some “credibility” to their feedback.

Let us forgive ourselves and one another: We are all going to make mistakes; so many of us are terrified that making a mistake means we are a racist, or sexist, or ableist, or transphobe, or ageist. In this field, being called those things when many of us are actively fighting for social justice cuts at the core of our identities. Even the thought of anyone even suspecting us to be any of those things hurts deeply. When someone makes a mistake, it does not mean they are a bad person. We have to forgive ourselves when we make mistakes, and we must try to forgive others.

Let us not give up on one another: We cannot build a strong community if we cannot converse with one another, have hard conversations, disagree once a while, and learn from one another. Let us stay and let us talk through the hard stuff. And if we do need to take a break, that is understandable, but let us leave the door open to come back.

## Abbreviations

IDEAS: Identity Development Evolution and Sharing
CFIR: Consolidated Framework for Implementation Research
AAQ-S: Acceptance and Action Questionnaire – Stigma
AIM: Acceptability of Intervention Measure
FIM: Feasibility of Intervention Measure
AIM: Appropriateness of Intervention Measure
TGD: Transgender and gender diverse
SUD: Substance use disorder
M: Mean
SD: Standard deviation
ACT: Acceptance and Commitment Therapy
CNA: Coincidence Analysis
ANOVA: Analysis of variance
